# Temperature-sensitive gating of TRPV1 channel as probed by atomistic simulations of its trans- and juxtamembrane domains

**DOI:** 10.1038/srep33112

**Published:** 2016-09-09

**Authors:** Anton O. Chugunov, Pavel E. Volynsky, Nikolay A. Krylov, Dmitry E. Nolde, Roman G. Efremov

**Affiliations:** 1 M.M. Shemyakin & Yu.A. Ovchinnikov Institute of Bioorganic Chemistry, Russian Academy of Sciences, ul. Miklukho-Maklaya, 16/10, Moscow 117997, Russia; 2 Joint Supercomputer Center, Russian Academy of Sciences, Leninsky prospect, 32a, Moscow 119991, Russia; 3 National Research University Higher School of Economics, Myasnitskaya ul. 20, 101000 Moscow, Russia

## Abstract

Heat-activated transient receptor potential channel TRPV1 is one of the most studied eukaryotic proteins involved in temperature sensation. Upon heating, it exhibits rapid reversible pore gating, which depolarizes neurons and generates action potentials. Underlying molecular details of such effects in the pore region of TRPV1 is of a crucial importance to control temperature responses of the organism. Despite the spatial structure of the channel in both open (O) and closed (C) states is known, microscopic nature of channel gating and mechanism of thermal sensitivity are still poorly understood. In this work, we used unrestrained atomistic molecular dynamics simulations of TRPV1 (without N- and C-terminal cytoplasmic domains) embedded into explicit lipid bilayer in its O- and C-states. We found that the pore domain with its neighboring loops undergoes large temperature-dependent conformational transitions in an asymmetric way, when fragments of only one monomer move with large amplitude, freeing the pore upon heating. Such an asymmetrical gating looks rather biologically relevant because it is faster and more reliable than traditionally proposed “iris-like” symmetric scheme of channel opening. Analysis of structural, dynamic, and hydrophobic organization of the pore domain revealed entropy growth upon TRPV1 gating, which is in line with current concepts of thermal sensitivity.

TRP cation channels are unique for their polymodal activation, contributing greatly to sensory physiology at different levels^1^. These modes include thermo-, pH- and mechano-sensation, nociception, taste, olfaction and vision^2^,^3^,^4^,^5^. Currently, there are 28 known mammalian TRP channels that are grouped into six sub-families (C, M, V, A, P and ML for canonical, melastin-related, vanilloid-binding, ankyrin repeat, polycystin, and mucolipin, respectively)^6^, with receptor member 1 of vanilloid-binding family (TRPV1) being the most studied structurally, biochemically, and computationally.

Thus, TRPV1 is the first channel from the whole TRP family that has been characterized structurally at high resolution, and one of the handful of channels that have both open and closed structures resolved^7^,^8^. Further, a plenty of biochemical studies have revealed the role of many residues in pH, ligand^9^ and temperature activation^10^,^11^,^12^,^13^. Moreover, it has been suggested that structural rearrangements that accompany activation by various stimuli differ as well, and the thermal sensing residues are located primarily (although not exclusively) in the pore domain^11^,^13^. Also, few advanced computational works already have inspected such important TRPV1 features as ion conductance^14^ and probable gating transitions^15^,^16^. Finally but probably not in the last, a body of pharmacological information has been collected for TRPV1 receptor^17^,^18^,^19^,^20^.

Given this data, one can attempt to uncover intimate molecular mechanisms of channel gating that would permit specific activation by chemical or physical stimuli, thus giving a clue for development of novel medicines^1^,^5^. For example, an experimental single-particle electron cryo-microscopy (EM) structures of one closed and two partially-activated states of TRPV1^7^,^8^ provide us with the knowledge that S1–S4 domains that are homologous to voltage-sensing domains in voltage-gated ion channels, remain relatively stationary upon activation (even upon ligand — capsaicin — binding to this domain). Major part of conformational changes accumulates inside the pore domain that is comprised of the channel selectivity pore filter and S6 transmembrane (TM) segment from each channel’s subunit. However, availability of these “extreme” structures and global thermodynamic framework for thermal^21^ and mechanical^22^ activation of the channel do not provide immediate understanding of the underlying gating mechanisms and pathways.

Additional insight into the molecular mechanisms of such phenomena can be gained if quantitative knowledge of the underlying dynamics and energetics of the channel is available. The latter one cannot be derived solely from the analysis of the static experimental structures of TRPV1. To proceed with this, atomic-scale structural/dynamic data coupling open and closed conformations of the channel *via* a series of intermediate states should be delineated under different conditions (heat, ligand binding, stress, and so on). Molecular dynamics (MD) is the method of choice to address the problem. Atomistic simulations are widely used in studies of a large variety of ion channels (see recent reviews)^23^,^24^,^25^,^26^. Moreover, several computational MD studies have been previously reported for the TRPV1 channel as well^14^,^15^,^16^,^27^,^28^. All of these studies have addressed different aspects of the receptor organization and action and, moreover, implemented different levels of approximation used to describe the complex protein-membrane system. Thus, in [14] the focus was put on conductance for Ca^2+^, Na^+^, K^+^ ions, and the channel was represented by the pore domain (without TM helices S1–S4) embedded into palmitoyloleoylphosphatidylcholine (POPC) bilayer. Apart from ion movements, exact conformational dynamics of the protein was described upon imposition of geometrical restraints on the receptor’s termini, including the lower gate (residues Ile 679–Lys 688). As a result, an interesting effect of asymmetrical response of the channel was observed. Zheng *et al*.^15^ simulated the full-length receptor (as taken from PDB) but in coarse-grained representation. This work was focused on normal mode analysis and delineation of collective motions involved in the TRPV1 gating transitions. The authors detected a quaternary twist motion of the TM domain relative to the intracellular part of the channel. Being interesting, the data do not explain with atomic resolution how the unrestrained gating proceeds because the accumulated intermediate states were generated *via* predefined progressive transformation of the protein between two extreme (O and C) states. Next, in the recent work by Poblete *et al*.,^16^ the whole TRPV1 immersed into POPC membrane was simulated with and without bound ligands – phosphatidylinositol 4,5-biphosphate (PI(4,5)P2) and capsaicin. The role of the latter ones in channel opening was investigated, especially in the lower gate vicinity. To accelerate the gating process, distance restraints were imposed on the crucial Ile 679 residue. It was shown that PI(4,5)P2 binding induces conformational rearrangements of the structure formed by S6 and TRP domains, thus leading to opening of the lower gate of TRPV1. Moreover, a model of human TRPV1 was built and used for MD calculations, and docking/screening of agonists and antagonists^28^. One of the important results is that different monomers change their conformations individually upon agonist/antagonist binding, thus implying asymmetry of channel function. Finally, recently a ligand-binding domain (S1–S4) of TRPV1 was simulated in a lipid bilayer along with capsaicin molecule to discover the role of membrane in binding site access by capsaicin^27^.

Despite these simulation efforts, the detailed spontaneous dynamics of the gating still remains elusive – just because the aforementioned studies were focused on other molecular aspects of the channel functioning (ion transport, role of ligands, effects of large-scale collective motions, and so on). As a consequence, the temperature effects were not taken into consideration in these works. To shed some extra light on the molecular aspects of the thermo-sensitive gating and to complement the previously published modeling data, here we compared in great detail open and closed states of TRPV1 channel pore and mapped hydrophobic properties of the pore inner surface, going far beyond initial assessment that was provided by the authors of these structures^7^,^8^. Further, we performed a series of full-atom MD simulations of both open and closed TRPV1 states (without N- and C-terminal intracellular domains) in explicit membrane at different temperatures, and observed partial temperature-dependent activation of the channel. Despite obvious limitations of dropping large cytoplasmic domains, these events provide us with probable mechanism of channel gating and the role of residues that might be important for temperature sensation and gating of the pore. Of course, like in many other macromolecular biological systems, neglecting of some (even distant from the point of interest!) parts may lead to the loss of important information about possible allosteric effects transmitted through the missing domains. This is especially true for the TRPV1 channel, whose activation is coupled to several protein regions^15^. That is why, the present simulation results were carefully examined for the existence of potential correlated motions of different TRPV1 parts and reproducibility of the results obtained in independent MD runs.

To summarize, here we describe a long-time MD study of TRPV1 full TM-domain with several functionally important juxtamembrane parts, like an extracellular loop 3 (which is absent from the experimental structures but might be involved in temperature sensation^13^), other loops, and TRP domain. We map pore radius profiles and hydrophobic properties of the pore inner surface along the entire MD trajectory, which is helpful in analysis of channels’ dynamics and properties, e.g. widely discussed “hydrophobic gating” effect^23^,^29^. Based on the accumulated exhaustive set of MD data (in total, c.a. 10 μs), we suggest an original mechanistic model of the temperature-dependent channel gating.

## Results

### Flowchart of the study

The background for this study is determination of TRPV1 channel structure in its basal (“closed”, C) and ligand-activated (“open”, O) states^7^,^8^. Here, we performed a series of atomistic molecular dynamics (MD) simulations of the channel in the explicit lipid bilayer starting from its both O- and C-states at different temperatures. The resulting MD data were further analyzed to resolve with atomic resolution behavior and properties of the pore region of the channel under different conditions.

The flowchart of the study is the following: 1) Assembly of two systems with O- and C-conformations of TRPV1 immersed into fully hydrated mixed lipid bilayers. 2) Calculation of series of 500- and 1000-ns MD trajectories for both systems at 280, 310, 325, and 340 K (for the full list of trajectories, refer to Table 1). 3) Analysis of TRPV1 dynamic behavior with focus on spontaneous opening of the pore at elevated temperature (initially closed state). 4) Analysis of hydrophobic properties of the pore in O/C trajectories. 5) Assessment of changes in solvation energy of the pore-lining residues in open/closed states extracted from the corresponding MD-trajectories. 6) Numerical processing of the complex dynamics of the pore domain aimed at vivid mechanistic interpretation of the molecular events upon gating. From these analyses, we draw conclusions on possible pore opening mechanism and probable role of “hydrophobic constriction” inside the pore. All these points are described in details below.

### Computational setup for MD simulations

Structures of the two TRPV1 states — closed (PDB Id: 3J5P^8^) and open (PDB Id: 3J5Q^7^) — were used for modeling. The whole TRPV1 in the membrane represents a very large system, which is currently being simplified in a long-time MD simulations by either cutting the size or reducing detail of representation. That is why, in all earlier simulation studies of the channel (see the references above), the authors were forced to make this compromise. The present work is not an exception. To reduce the size of the system and therefore to explore TRPV1 dynamics at microsecond timescales, cytoplasmic domains (with their ankyrin repeats) were omitted. By contrast, some other protein parts missing from the PDB models, including rather long extracellular loop 3 (ECL-3, residues 604–626 in the pore domain), were reconstructed (see *Methods* for details). As shown below (see Results and Discussion parts), the constructed model of TRPV1 seems reasonable because the omitted protein parts are rather distant from the pore – the area of our interest in this work. In addition, these domains are considered to be crucially important not for heat-operated gating, but rather binding of cytoplasmic partners, such as PIP2 or calmodulin^30^,^31^. Also, like in other computational studies of such mesoscopic systems, special care was taken to control reproducibility of MD results obtained in independent simulations, and the results were shown to be quite satisfactory.

**Figure 1:**
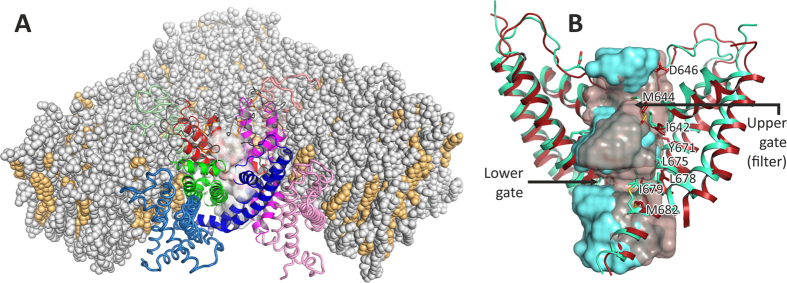
General view of modeled TRPV1 receptor in the membrane and comparison of open and closed structures. (**A**) TRPV1 (open state) in the model membrane (structure before MD). Each of the four receptor subunits is individually colored; pore domain is shown in brighter colors and in a *ribbon representation*. The pore void space is shown as *semi-transparent white surface*. The membrane consists of POPC/POPE (*gray color*)/cholesterol *(bright-orange)* mixture 2:1:1. Part of the membrane is removed for clarity. (**B**) A close-up comparison of pore domains of open (*red*) and closed (*cyan*) structures. S1–S4 domains and lipids are removed for clarity. Several important pore residues are subscribed. The pore void spaces are shown as *cyan solid* and *red* *semi-transparent surfaces* (closed and open structures, respectively). “Upper” and “lower” pore gates are subscribed; note almost full constriction of the pore in case of the closed structure.

These structures were immersed into hydrated mixed phospholipid/cholesterol bilayers (Fig. 1A). A set of MD trajectories at different temperatures were computed in the GROMACS suite: 1) open state (310 K; two trajectories: 500 and 1000 ns). Hereinafter, these trajectories will be referred to as O-310; 2) open state at 280 K (500 ns); O-280; 3) closed state at four different temperatures: 280 K (500 ns), 310 K (500 ns), 325 K (2 × 500 ns), and 340 K (500 and 1000 ns). Codes for these systems are C-280, C-310, C-325, and C-340, respectively; 3) extension of C-340 trajectory cooled back to 310 K (500 ns; C-340 → 310). Summary of the systems and description of the simulation protocol are provided in Table 1 and in the *Methods* section.

### Overall dynamics of TRPV1: flexible pore domain “anchored” with stable S1–S4 helix bundle

Inspection of the general behavior of all the systems in the course of MD simulations was carried out to check stability of the starting channel structures in O- and C-states under different conditions. The calculated overall properties, like root-mean-square deviations (RMSD) of TRPV1 coordinates from their starting values, root-mean-square fluctuations (RMSF) of the atoms demonstrated rather good equilibration and stable behavior of the protein tetramer and surrounding lipid membrane already after 100 ns of MD (Fig. S1A). As expected, the most conformationally labile parts of the receptor are found between the secondary structure elements, especially the reconstructed loop ECL-3 (Fig. S1B,C). Analysis of residues’ correlated motions in MD trajectories revealed that S1–S4 domain remains almost stationary and conformationally uncoupled from the pore domain (Fig. S2). Surprisingly, S1 motions are to some extent correlated with TRP helix movements. Therefore, in overall, the simulated large supramolecular system behaves reasonably well during long (~1 μs) MD runs – the tetramer is anchored to the membrane with its peripheral TM helical domains S1–S4, while the pore domain with neighboring extracellular loops is capable of fine-tuned conformational responses to a variety of external factors – at first, the temperature (see below).

### Structural/hydrophobic organization of the pore in the extreme experimental structures of TRPV1

In their original TRPV1 structures publications,^7^,^8^ D. Julius group has performed primary analysis of the pore domain conformation in open and closed states. In the present work, we proceed beyond these results by “mapping” of the pore radius and hydrophobic properties, with account of dynamic TRPV1 behavior. Superimposed structures of the pore domain in O- and C-states (starting structures before MD simulations) are shown in Fig. 1B; pore-lining residues that are discussed in the following sections are shown. In addition, a surface representation of the pore void spaces is presented. The principal distinct feature of the C-state is discontinuous pore volume with “upper gate” (the region of the selectivity filter of the channel) and “lower gate” (the region of the “hydrophobic constriction” formed mainly by the Ile 679 residue) apparently being the most narrow parts of the channel pore. In contrast, the O-state exhibits continuous void space along the whole pore axis.

**Figure 2:**
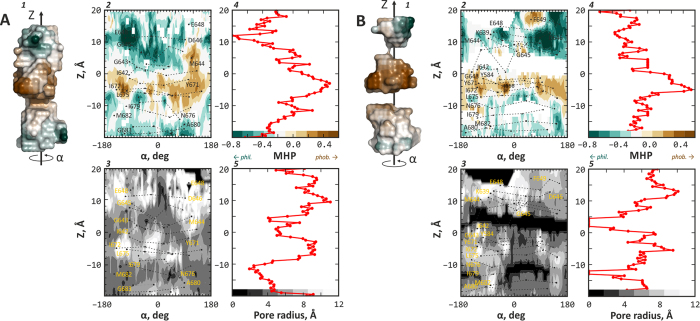
Detailed comparison of open (**A**) and closed (**B**) pores in TRPV1 structures. Each panel (**A**,**B**) contains five sections: 1) surface representation of pore void space colored according to the Molecular Hydrophobicity Potential (MHP) created by the receptor atoms (*celadon* is for hydrophilic and *brown* is for hydrophobic areas). Note the continuous pore for open (A1) and discontinuous — for closed (B1) structures. 2) *Upper left* section is a cylindrical map of MHP on the surface of the pore, where *Z* is the elevation along the pore axis and *α* is the rotation angle around this axis (see scheme in section (1)). MHP is color-coded; see the scale in section (4). Note white areas of MHP map in B2 due to lack of data (solvent-accessible surface points). 3) *Lower left* section analogously depicts the radius of the pore (color-coded according to the scale in section (5)). Sections (2) and (3) contain projections of several important pore residues; corresponding residues from different receptor monomers are shown with identical symbols and connected by *dotted lines*. 4) *Upper right* section shows MHP profile along the Z axis of the pore. Negative values correspond to hydrophilic areas, positive — to hydrophobic ones. The color scale above the MHP axis is used in sections (1) and (2). 5) *Lower right* section shows pore radius along its Z axis. Lower values (close to zero) correspond to the closed pore. The color scale above the radius axis is used in section (3). Note the difference between sections A5 and B5 (where “upper” and “lower” gates appear as zero pore radius in B5).

The main idea behind mapping of the pore inside TRPV1 channel is analogous to recently proposed in our laboratory the “Protein Surface Topography” approach,^32^ which postulates molecular surface of a protein to be the arena of intermolecular recognition. Here, grid points of the pore devoid of protein atoms are calculated (see *Methods*), and corresponding (semi)continuous surface (Fig. 2, panels *A1* and *B1*, show an example of TRPV1 open and closed starting structures, respectively) is analyzed in order to extract pore radius profile (Fig. 2, panels *A5* and *B5*) and protein hydrophobic/hydrophilic properties along the pore axis. These properties are estimated with use of the Molecular Hydrophobicity Potential (MHP) concept^33^ and computed with our Platinum software^34^ (see surface color coding in Fig. 2, panels *A1* and *B1*). Cylindrical projection of the surface shape or surface-distributed properties (here, MHP) around designated pore axis yields cylindrical maps of landscape properties or MHP distribution on the pore-accessible surface and axial distribution of the pore radius. For MHP, *celadon fill* shows hydrophilic and *brown* — hydrophobic areas of TRPV1 pore (Fig. 2, panels *A2* and *B2*). For pore radius, *black fill* is for close-to-the-axis surface regions and *white* — for off-axis (Fig. 2, panels *A3* and *B3*). Integration of the resulting 2D-maps over the cylinder rotation angle α provides 1D MHP profiles (Fig. 2, panels *A4* and *B4*) and radius profiles of the pore (Fig. 2, panels *A5* and *B5*).

This analysis reveals the following main specific and distinct features of TRPV1 O- and C-states (before MD simulations): 1) The pore contains a “hydrophobic belt” formed primarily by fours of Tyr 671, Ile 672, Leu 675, and Ile 679 residues, which come from four TRPV1 monomers (Fig. 2, panels *A2* and *B2*). This “belt” is highlighted by the maximum in the MHP profiles (Fig. 2, panels *A4* and *B4*). 2) The C-state is characterized by the two “bottlenecks” in the pore (Fig. 2*B2*), which may be attributed to the lower and upper gates of the pore and are rendered as black areas (zero radius) on the pore radius cylindrical map (Fig. 2*B3*) and corresponding minima on the pore radius profile (Fig. 2*B5*).

Next, we proceed with MD simulations of open and closed states of TRPV1 and analysis of the pore opening events that we encountered in these trajectories.

### “Opening” of the TRPV1 pore in MD simulations

**Figure 3:**
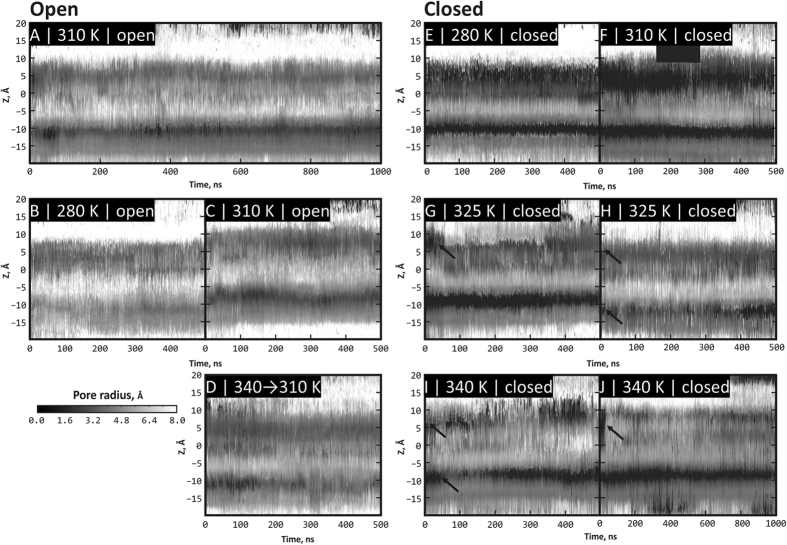
TRPV1 pore radius profiles in MD trajectories starting from open (**A**–**D**) and closed (**E**–**J**) states. In each panel, X axis is the MD time (500 or 1000 ns); Y axis is the elevation along the pore axis (**Z**). Actually, each panel is a time series of pore radius profiles (analogous to Fig. 2, panels A5 and B5), color-coded according to the scale in the bottom left corner. *Darker* areas correspond to closed regions of the pore (radius value around zero); *lighter* areas — to open regions. (**A**–**C**) Open pore at two temperatures (310 K/1000 ns, 280 K/500 ns and 310 K/500 ns trajectories, respectively). (**E**,**F**) Closed pore at 280 K and 310 K. (**G**–**J)** Closed pore at 325 K and 340 K (500 (×2) and 500 + 1000 ns trajectories, respectively). Arrows depict spontaneous opening of upper (**G**–**J**) and lower (**H**,**I**) gates of the pore. (**D**) Closed pore that was heated from 310 to 340 K (ending point in panel I) and opened, and subsequently was cooled back to 310 K, but remained stably open.

To assess the state of the pore during MD simulation, we calculated a “dynamic map” of the pore radius profiles (Fig. 3). On these maps, X-axis stands for MD time and Y-axis represents the pore radius profile along the Z-axis of the pore (e.g. Fig. 2*A1*) for a given MD snapshot. Each map is a time series of the pore radius profiles (exemplified by profiles in Fig. 2, panels *A5* and *B5*), color-coded according to radius of the pore. *Black* areas on these maps denote narrow regions of the pore that are stable over the simulation time, and *white* areas are wide regions that are not constricted by pore residues. Note closed regions that conform to upper and lower gates of the pore in the closed state of TRPV1 (Fig. 3E,F) and corresponding open regions in Fig. 3A–C.

The most intriguing effect was observed when the system initially taken in its closed state was heated above the activation temperature of TRPV1 (≈42–43 °C) to 340 K (67 °C; C-340) and 325 K (52 °С; C-325). It is seen that the upper gate (Fig. 3G–J) and sometimes the lower gate (Fig. 3H,I) open the pore, which is illustrated by the replacement of black band with lighter band on these maps (depicted with arrows). This effect might be not the complete opening of the pore, nevertheless water molecules start to pass the open gates. Probable activation mechanism should engage thermodynamic processes and conformational entropy changes of the channel. Thus, structural heterogeneity (and therefore configurational entropy) of the most flexible part of the channel in our model — extracellular loop 3 — in the open state of the channel is larger than in the closed one (true for several pairs of open/closed trajectories at the same temperature) (see Fig. S3).

At the same time, consequent cooling the system with partially open upper gate to the initial temperature (310 K) does not lead to the pore closure (Fig. 3D). Moreover, such closing was not observed in O-280 (7 °C; Fig. 3B) and O-310 (37 °C; Fig. 3C) trajectories, which were simulated from the very beginning at temperatures below the activation threshold for TRPV1 (≈43 °C) and therefore might have closed during the modeling. This may represent asymmetry of the “time arrow” that is an intrinsic feature of TRPV1 — according to the experimental observations,^35^ the channel closure proceeds much slower than the opening, thus making spontaneous channel deactivation practically unreachable in modern MD calculations.

One might hypothesize that TRPV1 channel opening in MD is a computational artefact and might be observed in any channel at elevated temperatures just for the reason of increased system mobility. To explore this possibility, we performed with the same protocol calculations of the closed state of KcsA potassium channel at 310 and 340 K. Apart from TRPV1, no evidence for even partial opening of this channel was observed (Fig. S4).

It is also useful to note that it is not only the channel itself but the bilayer membrane as well which serves the thermal sensor: repetition of the same experiment with heating the channel inside saturated and rigid dipalmitoylphosphatidylcholine (DPPC) bilayer did not result in TRPV1 opening (Fig. S5).

### Open pore in TRPV1 channel is more hydrophobic than the closed one

**Figure 4:**
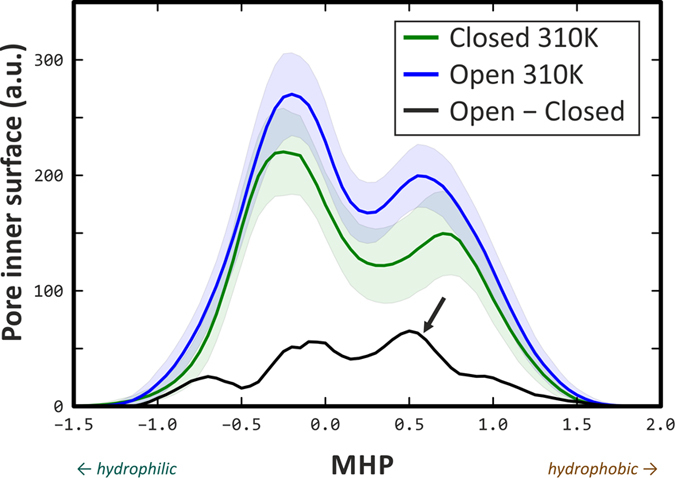
Open pore in TRPV1 is more hydrophobic than closed. Presented are MD-averaged MHP distributions (profiles) of the pore surfaces for open (*blue*) and closed (*green*) MD trajectories (O-310 and C-310, respectively). *Shaded areas* are standard deviations for MHP profiles from MD-averaging procedure. Distributions are not normalized, so the Y-values correspond to the pore surface area rather than to the probability density function; thus, greater area of the open pore can easily be noted. *Black line* is the difference between the open and closed MHP profiles; note an increase of the hydrophobic surface area (shown with an *arrow*). MHP values are given in logP units, where P is the distribution coefficients for water/octanol system.

Since the current theory of thermosensation in proteins requires that hydrophobic surface area changed substantially upon the conformational transition accompanying channel gating,^21^ we used our MD trajectories O-310 and C-310 to analyze the overall change of hydrophobic/hydrophilic surface area in the pore region. Figure 4 shows MD-averaged MHP-distributions for pore-lining residues in the closed (C-310; *green*) and open (O-310; *blue*) trajectories. The distributions are two-modal, indicating the presence of both hydrophobic (positive MHP) and hydrophilic (negative MHP) areas inside the pore. The distributions are not normalized, so the overall increase of the pore surface area is easily noted (*blue* curve lies all above the *green* one). *Black curve* shows the difference between open and closed MHP profiles (Open−Closed) and emphasizes overall increase of the hydrophobic part of the surface (shown with an *arrow*) – an integral taken over the MHP differential profile is positive. This result is consistent with the recently proposed thermodynamics consideration that thermally activated state should possess an excess of hydrophobic surface exposed to polar solvent^21^.

More detailed calculations revealed that the open state of the pore is more hydrophobic as if it exposed to solvent eight additional methylene (−CH_2_−) groups (details of the calculation are given in *Methods*). This is rather less than the aforementioned thermodynamic considerations require (one should not forget that we take into account only the pore region), but still confirms the tendency assumed: increase of hydrophobic surface area in the open state may contribute to temperature sensation. In total, using atomic solvation energy formalism,^36^ we identified a set of 23 TRPV1 residues that change their solvation energies between O- and C-states most substantially. Of these, only four line the pore surface: Gly 643, Tyr 671, Ile 679, and Ala 680, while other 19 are situated predominantly in the extracellular TRPV1 domain: Pro 456, Pro 461, Tyr 463, Lys 464, Asn 467, Arg 474, Gly 477, Lys 504, Lys 535, Gly 563, Ala 566, Arg 579, Val 595, Glu 600, Met 609, Glu 610, Ala 620, Thr 633, and Asn 652. Corresponding distributions of solvation energies for these residues are provided in Fig. S6.

**Figure 5:**
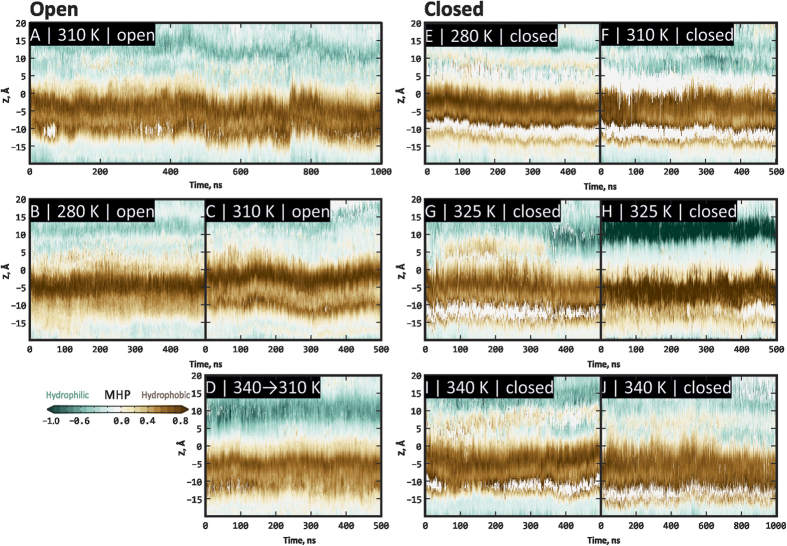
Hydrophobic properties (MHP) of TRPV1 pore in MD trajectories starting from open (**A**–**D**) and closed (**D**–**J**) states. This figure is analogous to Fig. 3, with the exception that the mapped property is not the pore radius but MHP. Each panel is a time series of MHP profiles (analogous to Fig. 2, panels A4 and B4), color-coded according to the scale in the bottom left corner. White “holes” in some maps (**E**–**J**) indicate insufficiency of data (when pore closes and no surface corresponds to a given Z value). For other details, see the legend to Fig. 3.

To assess dynamics and spatial distribution of hydrophobic/hydrophilic properties in the pore, we calculated dynamic maps of MHP profiles along Z-axis of the pore, analogous to the pore radius profile maps (Fig. 5). On these maps, time series of MHP profiles similar to those in the starting structures of TRPV1 are shown (Fig. 2, panels *A4* and *B4*). The “hydrophobic belt” formed by residues Tyr 671, Ile 672, Leu 675, and Ile 679 is clearly seen.

### “Piston-like” mechanics of the upper gate opening

Although both closed and open cryo-EM structures of TRPV1 are available, they do not give a comprehensive picture of channel opening during thermal activation. Moreover, the method of computational reconstruction of these structures from single protein particles involving imposition of four-fold symmetry may hide important structural and dynamic details of the activation process. At the level of the “upper gate” (selectivity filter), the most prominent difference between the experimental O- and C-states is the location of carbonyl oxygen atoms of Gly 643 residue, which is one of the principal determinants of the selectivity filter loop. In the closed structure, these atoms form a square with a side ≈3.9 Å and cross-sectional area of 15 Å^2^; open structure is characterized by doubling this area (30 Å^2^).

**Figure 6:**
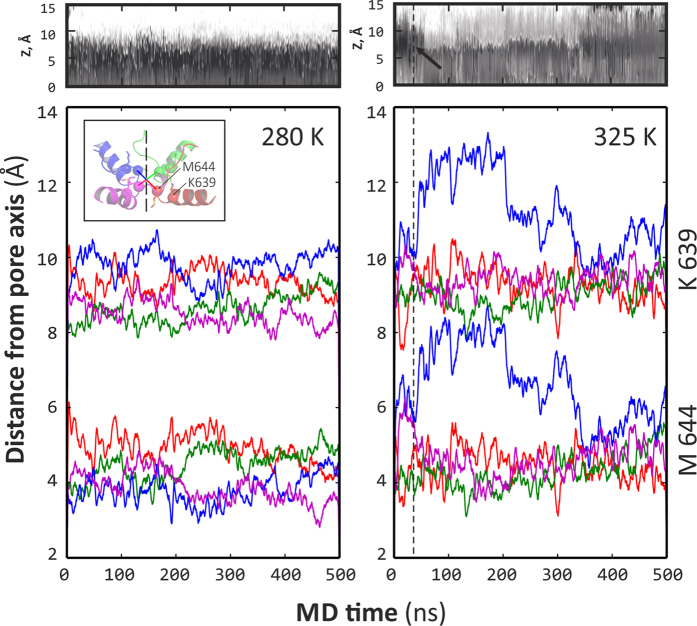
Collective and asymmetric nature of the opening of TRPV1 pore upper gate. The most pictorial geometric parameter that illustrates the difference between open and closed states of the upper gate is the distance of C_α_-atoms of the two key residues’ — Met 644 and Lys 639 — to the pore axis. Note that this is not the average distance for four protomers, but individual distances since pore opening seems stochastic and asymmetric process starting from fluctuation of a single pore filter segment. Here, these distances are shown for MD trajectories at 280 K (on the *left*), where no opening is observed) and 325 K (on the *right*), where one can note opening of the pore (see Fig. 3F). Plotted are aforementioned distances for Lys 639 (upper series of curves) and Met 644 (lower series). Any single curve in series stands for individual distance between residue C_α_-atom and pore axis. *Above* each panel a corresponding fragment of dynamic map of pore radius profile is provided (taken from Fig. 3, panels *E* and *G*, respectively). In *right* panel, an opening event is pointed with an arrow and dashed line.

**Figure 7:**
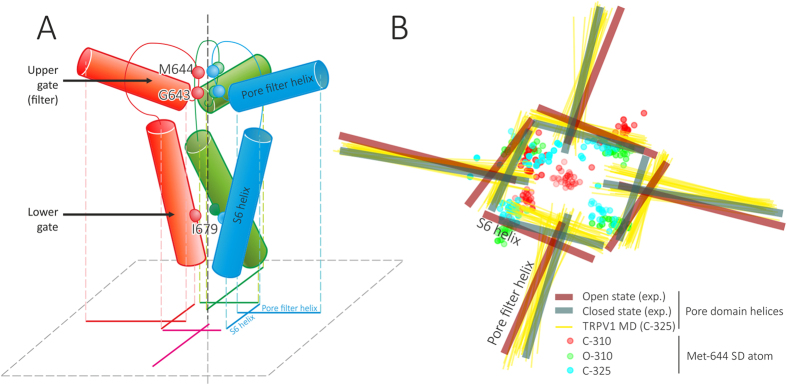
Geometry of the pore opening in TRPV1 channel. (**A**) Schematic representation of pore domain architecture and a projection that is used in panel (**B**) For each TRPV1 subunit (individually colored; the nearby one is omitted for clarity) two helices (pore filter (PF) and S6) and pore loop are shown. *Spheres* depict positions of several important residues: Gly 643 and Met 644 (upper gate) and Ile 679 (lower gate). A gunsight-like symbol is a projection of these eight helices (two from each subunit) on a membrane-parallel plane (*dashed gray*); two from one monomer are subscribed. Pore axis is shown with *vertical dashed line*. (**B**) Dynamic features of pore helices and Met 644 side chain in MD simulations. *Thick lines* show positions of TRPV1 pore helices (PF and S6) in closed (*teal*) and open (maroon) experimental structures. *Thin yellow lines* show positions of pore helices in C-325 MD trajectory. Circles depict projections of Met 644 SD atom in C-310 (*red*), O-310 (*green*) and C-325 (*cyan*) trajectories. Note that Met 644 SD atom from one of subunits blocks the pore in C-310 trajectory.

Surprisingly, in MD calculations there are almost no distinction in Gly 643 positions between O- and C-states, while the most striking difference that renders in pore closing and opening is manifested in the vicinity of Met 644 residue. In both “extreme” experimental structures, positions of C_α_-atoms of this residue are almost similar (the area of quadrangles built with the four C_α_-atoms ≈44 Å^2^), while in MD simulations this point becomes the most sensitive one in the upper gate opening. Thus, in the “closed” state (C-310 trajectory) this area is 30–35 Å^2^, increasing to 45 Å^2^ at 325 K (C-325 trajectory) and further to 55–70 Å^2^ at 340 K (C-340 trajectory). Immediate opening of the pore includes the clearance of the pore at the level of the upper gate from the lengthy side chain of Met 644 (Fig. 6). In MD simulations one of the most direct evidences of the “closed” pore is SD atom(s) of at least one of the four Met 644 residues that blocks the way (see Fig. 7B).

**Figure 8:**
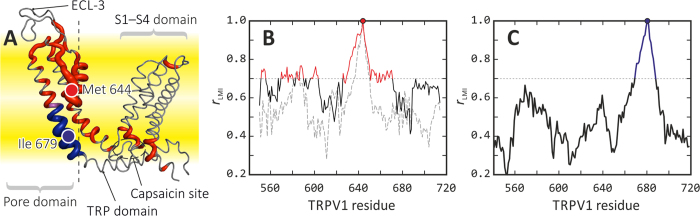
Correlated motions in TRPV1 “active” monomer during channel opening. (**A**) One of the four TRPV1 monomers that changes its conformation during channel opening most substantially (“active”), according to MD simulations. Residues moving synchronously (correlation coefficient *r*_LMI_ > 0.7, see *Methods*) with Met 644 (*red circle*), Ile 679 (*blue circle*) and both are colored *red, blue* and *magenta*, respectively. Sausage width shows maximum of two *r*_LMI_ values. Membrane is schematically shown with *yellow slab*. Pore axis is depicted with *vertical dashed line*. (**B**,**C**) Generalized correlation coefficient r_LMI_ was calculated using initial 100 ns of MD traces C-325 (**B**) opening of the “upper gate” at the level of Met 644 and C-340 (**C**) partial opening of the “lower gate” at the level of Ile 679. Values above 0.7 are colored *red* (**B**) and *blue* (**C**) This data was used to map and color TRPV1 monomer in (**A**). Note that highly-correlated with Met 644 regions (**B**) are found only in the “active” monomer (*solid line*) but not in any other monomers (*broken line* depicts one of the three). Curves in B and C panels are sections of full correlation map shown in Fig. S2.

Furthermore, detailed inspection of possible collective moves in the pore domain of individual TRPV1 subunits revealed an unexpected and interesting picture of the upper gate mechanics. Thus, increase of the temperature ensures more conformational freedom of only one (!) of the pore filter helices (residues 630–639), which finally results in a relatively large shift of the whole helix away from the pore axis for at least 3 Å. Such a “piston-like” movement of the helix pulls the pore loop (residues 640–644) in the same direction and frees the pore (see Fig. 6). As shown in Fig. 8, the movement of C_α_-atom of Met 644 in this protein subunit is highly correlated with residues 626–670. By contrast, in other subunits the collective motions are limited to a much smaller region (e.g., 635–650) (Fig. 8B). By other words, relatively large part of one of the four pore domains — from the end of ECL-3 (residue 626) up to the middle of TM helix S6 (residue 670) — spontaneously undergoes a large-scale collective displacement (Fig. 8), *red color*), which results in partial opening of the channel in the upper gate. Much smaller movement is detected in another subunit, while the two rest ones stay almost fixed.

### Correlated motions of S6 and TRP helices upon the lower gate opening

At the same time, the picture of collective motions at the level of the lower gate (Ile 679 and its neighbors) is rather different from that observed for the upper gate (compare Fig. 8B,C). In MD trajectories that feature partial opening of the lower gate (C-325.2 and C-340.2), the correlation coefficient with Ile 679 motions is high (*r*_LMI_ > 0.7) only for the part of S6 helix — residues 670–685. Moreover, it’s almost similar for all — “active” (moving in the pore opening process) and “non-active” subunits. This demonstrates that the opening of the lower gate is regulated by rigid-body movement of the helix S6.

The commonly accepted major determinant of the lower gate is residue Ile 679, which significantly changes its conformation and solvent accessibility upon the C → O transition. The “hydrophobic belt” of Ile 679 and other non-polar residues that jam the pore in the closed structure uncouple and form a channel with non-polar walls (see Fig. 2*A2*), where water and ions can pass through. The area of the square, built using four C_α_-atoms of Ile 679, is respectively 50 and 90 Å^2^ in the experimental closed and open structures. In the course of MD simulations, these areas fix themselves relatively stationary at levels 50–60 Å^2^ and 80–110 Å^2^, respectively. Moreover, in a half of MD trajectories at elevated temperatures we observe partial opening of the lower gate (C-325.2 and C-340.2). As seen in Fig. 3H,I, partial opening of the lower gate is observed (marked with an arrow) — the corresponding area of the aforementioned quadrangle formed by C_α_:Ile 679 grows from 55 up to 75 Å^2^ (value for C-340.2). Like in the case of the upper gate, the movements of TRPV1 subunits are not synchronous – the major role is played by just one of them.

At the C-terminus, helix S6 is connected *via* a short linker with another helix – the so-called TRP domain. To check possible coupling between movements of these two helices, the degree of bending of this region was calculated for all MD trajectories using the Bendix program^37^. Resulting plots of the bending angle *vs.* MD time for closed, open, and partially open (*via* lower gate) states are shown in Fig. S7. It is seen that in the C-conformation the S6–TRP region is broken only in the linker (685–693), while the O-state reveals additional helix kink on residue 704. Interestingly, partial opening of the lower gate at T = 340 K and 325 K is accompanied with appearance of the similar kink (704) for one of the TRPV1 subunits (Fig. S7, panels D and F, respectively). It is important to note that this is the same protein subunit, which liberates the pore at the Ile 679 level (see above).

## Discussion

Although method of molecular dynamics (MD) comes of age, and many large molecular systems such as ATPase, ribosome or viral capsids may be modeled at a reasonable timescale^38^, it’s still somewhat an art, to discover novel biological information from mechanistically-determined computational models. Given this, it’s a kind of fortune to observe in the presented mesoscopic multi-component system such a fine effect like protein thermal sensitivity, which results in a certain conformational event — spontaneous (partial) opening of the pore in the TRPV1 ion channel. This success might result from several favorable circumstances.

First, our TRPV1 model is rather complete, although the reader should be aware that we intentionally dropped rather large and flexible cytoplasmic domains to keep the system size reasonable, thus the model cannot be treated as ultimately accurate. The omitted features are ankyrin repeat domain with the following linker, and C-terminal domain: currently, in most MD simulations of large systems, it’s compulsory measure to simplify the system by cutting its size^14^,^27^ or reducing detail of representation^15^. Experimental works confirm that thermal sensitivity, at least in part, is localized in the pore domain^30^, while truncated domains certainly contain binding sites for PIP2 and calmodulin^31^, which are not studied in our work. Still, the model contains TRP-domain, which is important for receptor activation^10^, and extracellular loops (ECL) 2 and 3, which were not resolved in experimental structures^7^,^8^. There is slightly controversial data on the role of ECL-3 in thermal activation of TRPV1^13^,^39^, however a large body of biochemical data still confirms its importance for various modes of receptor function^11^,^12^,^30^,^40^. Hence, reconstruction of this part of the protein seems to be worth the effort. Indeed, our MD-based mechanistic model of the upper gate opening confirms the pivotal role of ECL-3: (i) its configurational entropy is larger in O- *vs.* C-state (Fig. S3), although we treat with caution particular conformations of ECL-3 and do not claim that the structure is exactly as in our model; (ii) this loop is directly linked with the extended movable pore filter, which undergoes a “piston-like” shift clearing the upper gate of the channel (Fig. 6).

Moreover, including into the model of the TRP-domain was also important because its dynamics is strongly coupled with the lower gate mechanics and, therefore, shed new light on possible allosteric effects propagating from the ligand binding site to the pore. Many studies declare that major part of the thermal sensor of TRPV1 is the pore itself^12^,^41^, and some previous MD studies have included only this part of the protein^14^. We therefore realize that spontaneous channel opening is only detectable if all the aforementioned neighbors of the pore domain are taken into account in MD simulations. Finally, the model includes TM helices S1–S4 with the related loops: being fairly distant from the pore, these TM domains provide overall stability of the channel in the membrane (see *Results* and Fig. S2) and its coupling to the lipid environment, which seem to be very important for TRPV1 activity.

We believe that the relevance of the TRPV1 model for the studied problems is additionally proved by the fact that the detected effects of the channel opening are determined by concerted motions of just a few clearly positioned structural elements of TRPV1 in the pore domain and its neighboring regions. According to our MD-data, these parts are rather “mechanically independent” – their coupling to the peripheral domains is not large enough to change significantly the picture of the pore opening/closure. By other words, the pore domain has the own set of normal modes, which determine its possible movements (including high-amplitude ones), while external factors can mainly trigger and/or tune the pore dynamics.

Second, thermal sensor is not only the protein itself, but also biomembrane where the channel is immersed into. We chose a three-component mixed phospholipid / cholesterol bilayer, which mimics neuronal membrane with respect to its lipid composition^42^. Several studies on TRPV1 in artificial membranes show its normal function when using similar phospholipid composition, although additionally these experiments revealed versatile role of phosphoinositides in activation of the channel^43^,^44^,^45^. This choice might have played an important role, since if membrane medium was chosen to be saturated and rigid DPPC bilayer, not a single TRPV1 pore opening event has encountered in our simulations (Fig. S5). On the other hand, being liquid in the whole range of studied temperatures, the membrane used in our simulations slightly changed its properties, thus facilitating temperature-dependent channel gating (to be published). We should note that the exact role of membrane lipids in regulation of TRPV1 is still not well understood. Although some authors suggest that the mechanical properties of lipid bilayer affect the channel functioning^46^,^47^, measured temperature- and ligand-response profiles of TRPV1 reconstituted in liposomes were not changed as compared with cell membranes^43^, thus permitting a conclusion about insensitivity of the receptor to properties of surrounding lipids. We should note however, that the latter experiments were carried out in rather “fluid” liposomes (containing POPE, POPC, POPG (palmitoyloleoylphosphatidyl-glycerol), Chol and SM (sphingomyelin)), while we performed one “control” MD simulation in much more rigid DPPC bilayer (Fig. S5). Hence, one can assume that the channel works correctly in liquid membranes of various composition, although serious augmentation of lipids viscosity can “freeze” the gating.

Third, the choice of the force field for MD simulations also might be the critical issue^48^. We used modern all-atom version of Amber force field supplemented with one of the latest GROMACS versions that should be accurate enough to account fine temperature and solvation effects. The force field also contains Slipids addition^49^ that increases accuracy in modeling of phospholipid bilayers, probably including their thermal response.

Fourth, our simulations were rather lengthy and lasted at least 500 ns (up to 1000 ns; ≈10 μs in total). It is far behind the best modern practices, which employ latest supercomputers^50^,^51^,^52^, however our work still demanded very high computational power. As a result, we were able to observe such biologically relevant events like opening the pore in the ion channel. It is noteworthy that in several independent lengthy MD runs we observed very similar phenomena related to “activation” and pore opening. High reproducibility of the results proves adequacy of the selected model. In addition, our MD data are not contradicting to known experimental information. Here, we would like to stress out that all the simulations were carried out without any imposed restraints, which previously have always been used in MD simulations performed to explore other dynamic aspects of the TRPV1 channel behavior^14^,^16^).

Summarizing, despite obvious limitations attributed to the lack of N- and C-terminal cytoplasmic domains, our contribution provides an interesting insights on TRPV1 function, expanding findings of previous structural and computational works.

There are many interesting effects in the modeled system that are beyond the scope of this article, such as details of membrane influence on the thermal sensitivity and effect of solvation and water/ions passage through the channel; this work will be published elsewhere. In this contribution, we focus primarily on the pore domain and its closest neighbors, although we aware of restrictions of such a “tunnel vision”.

Maping protein channel’s pore in the dynamics — namely, its radius profile and hydrophobic properties —permitted accurate assessment of TRPV1 opening events that are encountered in our simulations at elevated temperature (325 K and 340 K; both above TRPV1 activation threshold). As it is seen from Fig. 2G–I, TRPV1 pore in our simulations efficiently opens at the level of the upper gate, which is not the complete opening and may be considered as a starting event that would finally lead to fully open state of TRPV1. At the level of the lower gate, openings seem to occur more rarely (in two trajectories at elevated temperature of four total), suggesting higher activation barrier for this event. It is important to note that we did not encounter channel closing events. Neither cooling the system back from 340 K to starting temperature 310 K (Fig. 2D), nor MD calculations starting from the open conformation at temperatures that are below the channel activation threshold (Fig. 2A–C) did not result in a single closing of the channel. This might result from asymmetry of channel’s opening kinetics:^35^ rapidly opening, it might have require much longer times to “settle down” and close the pore once the temperature decreases.

The opening of the pore at level of the upper gate is a sequence of events when single pore filter elements from different TRPV1 subunits move away from the pore axis, freeing the way for water and ions. The important feature that is not seen from the open experimental structure is asymmetry of this process: in cryo-EM experiment a four-fold symmetry is imposed in the structure calculation procedure, therefore such fine details elude the eye of the experimentalists. Thus, Fig. 6B shows how at 325 K one of the monomers (*blue curves*) starts moving in a distal direction, increasing distance between the watched residues (Lys 639 and Met 644) and the pore axis. This is rather low-amplitude (≈2–3 Å) but a highly correlated movement (correlation coefficient between two blue curves is ≈0.95; see also Fig. 8), which is not observed at low temperature (Fig. 6A). (This result is provided for trajectory C-325.1; for C-325.2 we observed similar picture.) Simultaneously, Met 644 side chain shifts away from the pore void space, where it resides at low temperature (Fig. 7B, *red markers*). Note that other monomers remain relatively stationary in most MD runs. It’s remarkable that the moving part of the “active” monomer is rather large — it includes residues from 626 to 670.

The aforementioned “piston-like” movement of the protein region forming the upper gate of TRPV1 was the most prominent and well reproduced result extracted from MD data. By contrast, simulation results related to opening of the lower gate are rather scarce – we succeeded in detection of only two such events in the trajectories C-340.2 and C-325.2 (of total four trajectories where upper gate opens). Anyhow, detailed analysis of the dynamic data permitted elaboration of an interesting mechanistic picture of the partial channel opening in the lower gate: movement of the helix S6 accompanied with bending of the region S6–TRP and destabilization (bending) of TRP helix on residue 704. It is remarkable that the movement of the crucial residue Ile 679 regulating opening of the lower gate is correlated with only a short fragment of the helix S6 (residues 670–685), thus being independent of the upper gate “mechanics”. Residue Ile 679 seems to be one of most important for lower gate function (not least because if forms a “hydrophobic belt”), although its substitution to Ala does not disturb channel function, but rather removes mutual potentiation with activation by capsaicin^41^. Most likely, simultaneous mutation of adjacent residue(s) or replacement of Ile 679 with a more voluminous residue (e.g., Phe or Trp) would alter gating process as well.

Also, it is noteworthy that TRP helix is located near the cytoplasmic face of the helix bundle S1–S4 and lies in the close proximity of the ligand-binding site for capsaicin^7^. In some sense, TRP domain resembles a “cardan shaft” in a motor-car, which transmits structural changes from the distal ligand site to the channel’s pore. Hence, we suggest that the conformational transition observed in our MD simulations for rather complete and representative model of TRPV1 channel gives for the first time an intriguing mechanistic view on the molecular events coupling the pore domain and other remote functionally important sites. Taken together, the elaborated model for pore regulation in both upper and lower gates represents a mechanistic gear with two independent movable parts, which sense the temperature and ligand binding *via* “belting” formed by specific backbone regions located between the C-terminus of ECL-3 and TRP domains.

One of the crucial results of this study is the found asymmetry of the channel gating. Unlike the ideally symmetric experimental EM-structures of TRPV1 and the “iris-like” mechanisms of gating proposed earlier for some ion channels (ref. 53 and references therein), the detected movements of the protein subunits are not synchronous — the major role is played first by only one of four monomers. Similar effects were previously detected in experiments for mechanosensitive channel MscL^53^. Some asymmetric aspects of subunits’ behavior (although not the gating) were also observed upon modeling of TRPV1^14^,^16^,^37^. Taking into account the mechanistic nature of our MD-based model, it is reasonable to propose that such an asynchronous “engine” is more efficient in terms of speed and robustness than the fully symmetric one. This seems to be especially true for the initial stage of the channel activation – in order to shift the equilibrium from C- towards O-state it is sufficient to rattle just one monomer. Selection of such a “weakest link” can occur stochastically. Altogether, one can conclude that the observed asymmetry may be a common molecular principle of opening of multimeric channels.

Unfortunately, MD-statistics for opening of the lower gate (just two events) is not enough yet to affirm the proposed mechanism. To justify it, it is necessary to observe multiple such events in MD, e.g., by increasing sampling far beyond 1 μs, which is possible with the latest supercomputers but still hardly reachable with most of the current systems. Also, even for the upper gate we are capable of observing only the very initial phase of the channel opening. The real gating mechanism should employ subsequent movements of several monomers, yielding progressive opening of the pore.

But what are the driving forces of temperature sensitivity of TRPV1 channel, which result in opening of the pore that is described above? Many biochemical studies aimed to localize a certain protein domain that is in charge of this function^4^,^5^,^13^,^35^,^39^, but, contrary to voltage sensing in voltage-gated ion channels, the picture remains vague. Thermodynamic considerations state that “the source of high temperature sensitivity is a difference in heat capacity [(ΔC_P_)] between channel’s open *vs.* its closed conformation”^21^. “Large” ΔC_P_ means the order of several kcal/mol-K. Basic calculations show that such effect can be achieved by exposure of ≈300 hydrophobic (methylene) carbons to water upon activation (opening), which is approximately equivalent to 10–20 nonpolar side chains per TRPV1 monomer^21^. Following this, heat-activated proteins should have positive enthalpy change (ΔH° > 0), while cold-activated — negative. Similarly, for heat-activated proteins ΔS > 0^13^,^21^.

Since we have MD trajectories of TRPV1 in both closed and open states (C-310 and O-310, respectively), we asked if such change in exposure of hydrophobic groups is detectable from the simulations. We used two complementary approaches that are widely used for assessment of hydrophobic properties of biomolecular systems — formalisms of the Molecular Hydrophobicity Potential (MHP)^33^ and the Atomic Solvation Parameters (ASP)^36^,^54^. Using a set of residues that form a pore, we calculated over MD trajectories the profiles of MHP distribution on the solvent accessible surface of the pore (Fig. 4). It was shown that open pore residues are more accessible to solvent, and their total hydrophobic surface area is larger (denoted with an *arrow*). This is in line with aforementioned thermodynamic considerations, and to proceed with more quantitative approach, we estimated that this change could be attributed to solvent exposure of just 8 methylene groups (see *Methods*). This is not much, as compared to the requirement of 300 such groups^21^, however, given that our focus is just the pore-lining residues, this finding confirms the existence of the effect. However, such residues responsible for ΔC_P_ changes most probably are widely distributed over the whole TRPV1 molecule, and many other residues, apart from the pore-lining ones, might participate.

To extend this observation, we used the atomic solvation parameters formalism to delineate TRPV1 residues that change their solvation energy (*E*_solv_) most significantly between closed and open states dynamics. As a result, four pore-lining residues and 19 residues from other regions were found (Fig. S6). These may be “hotspots” for temperature sensing in TRPV1 in the TM-domain. Most of the detected residues (Tyr 671, Ile 679, Ala 680) form the “hydrophobic belt” inside the open pore, which is clearly seen in the pore MHP maps (Figs 2A and 5A–C). Taking together, our results show that hydrophobicity of the pore surface increases upon gating. In another mechano- (MscL)^53^ and voltage- (KscA) sensitive channels (both are not thermosensitive!) such effect is not observed (Fig. S4B,C). This might serve an additional indication of an interesting specific feature of the thermal activation of TRPV1 – its opening results in strengthen of the hydrophobic gating.

As discussed above, increased hydrophobicity of the pore upon channel opening contributes to the entropy growth. But this might be not the only source of positive ΔS that accompanies C→O transition. Additional gain of entropy can be achieved due to the changes in the conformational flexibility of the system. Again, we do not intend to accurately account for the whole conformational entropy change — e.g., lipids, water and large peripheral protein parts are not taken into account. Instead, we tried to find some evidence that ΔS has a positive sign, which should characterize heat-activated protein: the most flexible TRPV1 part in our model — the loop ECL-3 — exhibits more conformational freedom in the open state (Fig. S3), which obviously should contribute to thermal sensitivity. This is definitely not just the temperature effect that increases average protein flexibility: the observation is made from C-310 and O-310 trajectories that were computed at the same temperature (two sets of trajectories: C-310.1/O-310.1 and C-310.2/O-310.2). There is an experimental evidence that ECL-3 fragments from different monomers should come closer upon TRPV1 opening^13^, which becomes possible due to the increased conformational heterogeneity of these loops. Additional contribution to the positive ΔS upon C → O transition can be given by extra bending of the S6–TRP region (see above).

Just as in case of analysis of geometric aspects of TRPV1 pore opening, we chose potassium channel KcsA as a negative control: analogous calculations with closed and open states of this channel did not show significant change of pore surface hydrophobicity and solvation energy (Fig. S4). This additionally confirms that only temperature-activated proteins like TRPV1 possess large enough change of heat capacity (ΔC_P_) and corresponding change of *E*_solv_.

From our results, role of several residues that have not been assayed previously, can be drawn. First, Gly 643 and Met 644 that form the upper gate (see geometry of the pore opening in Fig. 7), should play pivotal role in ion conductance. Most probably, G643A mutation would inhibit conductance; M644A and M644L mutations, as shown previously, increase and decrease conductance for large organic cation NMDG, respectively^40^, confirming its boundary position inside the selectivity filter. Second, Ile 679, which forms hydrophobic constriction of the lower gate, should also be responsible for ion conductance. Although I679A mutation exhibited unchanged temperature activation profile^41^, we suggest that double mutation I679A/A680G should produce phenotype with “always open” lower gate. Third, Asn 676, which interacts according to our preliminary results with intracellular Ca^2+^ ions, may be in charge of process of desensitization, and N676S/T mutation may produce phenotype with altered desensitization parameters. Fourth, mutations of Lys 688 may alter mobility of TRP domain: K688G likely will increase mobility, probably increasing channel sensitivity to activators, and K688P, on the opposite, may decrease it. Although, these are only preliminary assumptions, validation of which is a subject of separate work.

## Conclusion

In this work, we performed unrestrained atomistic MD-simulations of TRPV1 ion channel embedded into a mixed phospholipid/cholesterol bilayer membrane. A set of MD runs starting from the experimentally determined closed (C) and open (O) states of TRPV1 was carried out at temperatures 280 K, 310 K, 325 K, and 340 K, thus providing rather good sampling below and above TRPV1 temperature activation threshold (43 °C or 316 K). The most interesting and somehow unexpected phenomenon observed in these computational experiments was temperature-dependent opening of TRPV1 pore at the level of the “upper gate”, which corresponds to the selectivity filter of the pore. Also, in two MD runs at T = 325 and 340 K partial opening of the “lower gate”, where several non-polar residues form a hydrophobic constriction, was detected. Detailed analysis of the voluminous body (~10 μs) of dynamic data permitted elaboration of an original mechanistic model of TRPV1 gating within the pore domain. The model consists of two largely independent movable parts, which sense the temperature and (potentially) ligand binding *via* a “belting” formed by particular backbone regions from the C-terminus of the extracellular loop ECL-3, pore and TRP domains. It was shown that opening of the upper gate is regulated by a collective “piston-like” movement of the pore filter and neighboring regions (residues 626–670), while permeability through the lower gate is guided by the correlated motions of the helices S6 and bending in TRP domain. In turn, these two dynamic determinants are strongly coupled to their neighbors – flexible loop ECL-3 and the helix bundle S1–S4, respectively. Such a mechanistic view explains two-stage gating and putative molecular origin of local and allosteric conformational transitions determining TRPV1 channel functioning.

Unprecedented “resolution” provided by the computational experiments helps to understand atomic picture of early events that precede full-scale pore opening: this is asymmetric movement of pore filter segments in the distal direction away from the pore axis. In both upper and lower gates, this process necessarily starts with one sole TRPV1 monomer, and others may join at the later stages. Regardless of the fact that similar effects were observed in mechanosensitive and some other channels (including TRPV1), this finding is not common: most experiments on multimeric ion channels produce symmetrical structures that imply that opening progresses also in the symmetric way. It seems reasonable that such an asynchronous “engine” suggested for TRPV1 is faster and more robust than the fully symmetric one, especially at the initial stage of the channel activation – in order to shift the equilibrium from C- towards O-state it is sufficient to rattle just one monomer.

The collected structural and dynamic data were complemented with the advanced technique for pore mapping with respect to its radius and surface hydrophobic/hydrophilic properties (for more details see Methods and Supplementary Information). The results reveal a hydrophobic “plug” which becomes a “belt” upon opening of the lower gate. This unburies a considerable hydrophobic surface area and is accompanied with growth of the conformational lability of protein regions bordering the pore domain. Altogether, this increases entropy of the system upon gating and along with other molecular events appears to be one of the driving mechanisms of temperature sensitivity of TRPV1.

Summarizing, in several important aspects (asymmetric behavior, coupling of TRP helix bending and pore opening, etc.) our computational findings agree well with the earlier independent observations. Moreover, they complement the previously published experimental and modeling data on structure and dynamics of TRPV1. Thus, they shed some new light on early steps of TRPV1 activation due to temperature increase — spontaneous conformational rearrangements in the TM region. Taking into account reduced size of the computational model, we assume that the observed effects can represent only a part of a more complex TRPV1 gating, which could also be allosterically coupled to some distal domains (including ankyrin repeats, and so on). Ongoing research based on this may further clarify this picture.

## Computational Methods

### Construction of the systems

3D structures of the two TRPV1 states — closed (C; PDB Id: 3J5P^8^) and open (O; PDB Id: 3J5Q^7^) — were used for modeling. To reduce the system size, cytoplasmic domains (with its ankyrin repeats) were omitted, thus the simulations were performed for the tetramer of channel fragments with residue numbers 427–719. Two missing loops in this range that are absent from the experimental structures were reconstructed: the short one (residues 503–507) using MODELLER^55^ and the more lengthy one (residues 604–626), which is probably the part of the temperature sensor, — by pyROSETTA using loop reconstruction protocol^56^.

The initial configurations of the simulated systems were obtained by the insertion of protein into the pre-equilibrated lipid bilayer comprised of 400 molecules of palmitoyloleoylphosphatidylcholine (POPC), 200 molecules of palmitoyloleoylphosphatidylethanolamine (POPE) and 200 cholesterol molecules (CHOL) using *genbox* utility from the GROMACS package. The latter procedure leads to removal of a number of lipid molecules to avoid atomic clashes. Water molecules were added to the simulation box, and those located inside the bilayer were removed from the system. Finally, chloride ions were added to restore the electroneutrality. Thus prepared systems were equilibrated by energy relaxation using the following protocol. 1) 5 × 10^4^ steps of steepest descent minimization followed by 2) heating from 5 K to the target temperature (280, 310, 325 or 340 K) during 100 ps MD run and 3) 5 ns of MD run at given temperature with fixed positions of the protein’s atoms to permit membrane relaxation after insertion of the large transmembrane protein. Finally, long (500 or 1000 ns) production MD runs were carried out for each system. Details of the performed MD calculations are presented in Table 1.

### Molecular dynamics protocol

All MD simulations were performed using the GROMACS 4.5.6 package^57^ and Amber99sb-ildn forcefield^49^. Simulations were carried out using integration step of 2 fs and imposed 3D periodic boundary conditions. A twin-range (10/12 Å) spherical cutoff function was used to truncate van-der-Waals interactions. Electrostatic interactions were treated using the particle-mesh Ewald summation (real space cutoff 10 Å and 1.2 Å grid with fourth-order spline interpolation). MD simulations were carried out in the isothermal-isobaric (NPT) ensemble with a semi-isotropic pressure of 1 bar and a constant temperature (individual for separate runs). The pressure and the temperature were controlled using the V-rescale thermostat^58^ and Parrinello-Rahman barostat^59^ with 1.0 and 0.1 ps relaxation parameters, respectively, and a compressibility of 4.5 × 10^−5^ bar^−1^ for the barostat. Protein, lipids and water molecules were coupled separately.

Helix bending angle for S6–TRP channel segment was calculated according to Bendix method^37^ using VMD Bendix plugin.

### Mapping of the radius and hydrophobic properties of TRPV1 pore

In this work, we used a custom algorithm for mapping TRPV1 pore radius and hydrophobic properties along the pore axis. First, we detected cavities in protein from the analysis of 3D-density grid with the cell size 3.4 Å. To account for regions of possible pore closing, the cavities intersected in the membrane plane (xy-plane), were merged; the largest cavity was considered as a pore. Then, the dot Сonnolly surface of TRPV1 was calculated with dot density 3 per Å^2^. Points of the surface located at grid cells of the pore cavity and adjacent cells form the pore border (set S). Then, the pore along the membrane axis was characterized at the range −20..20 Å from the bilayer center with a step dz = 0.5 Å. At the first stage, we selected from subset S the points located between z ± dz (subset S_z_). Center of the pore C(z) was defined as a mean point from S_z_. The mean distance from the pore center to points on the border (from S_z_) was taken as a pore radius R(z). Hydrophobicity of the pore H(z) was equal to the average hydrophobicity of points from Sz. These values were calculated for each MD frame and presented as colored contour map (z, t) for hydrophobicity and gray map for pore radius.

Properties of the pore for a single MD frame were presented as maps (α, z), where α is the rotation angle along the pore axis and z is the shift along the membrane normal. For generation of these maps, coordinates of the points from the subset S were converted to (α, z). This method is somehow similar to the wide-spread HOLE program^60^, although it’s more flexible and suitable for programming pipelines. The main advantages of the method are: 1) account for dynamic pore behavior; 2) calculation of distribution of physico-chemical properties over pore surface; 3) mapping of the pore as a cylindrical map (for static structures) or pore radius/MHP profiles (for MD trajectories). The performance of our mapping method in comparison with HOLE (on the example of pore radius profile) is provided in Fig. S8.

### Assessment of the residues’ solvation energy and thermodynamic quantities

Solvation energy of residue *i* was calculated as follows: *E*_solv._(*i*) = ASP(*i*) × ASA(*i*), where ASP(*i*) and ASA(*i*) are the atomic solvation parameter (taken from ref. 54) and the solvent accessible surface area of atom *i*, respectively.

Estimation of the heat capacity (ΔC_P_) change upon C → O transition was done based on the difference between the average total MHP values calculated in the solvent accessible surface points for C- and O-states. MHP values were averaged over equilibrated parts of the corresponding MD trajectories (C-310 and O-310). Using small model compounds (hexane, pentane, etc.), it was shown that the sum of surface MHP for one methylene group is equal to c.a. 50 MHP units. Being calculated under the same conditions, the total MHP difference between C- and O-states of TRPV1 was estimated to be 400 MHP units, thus corresponding to the exposure of 8 additional −CH_2_− groups in the open state. According to [21], transfer of one nonpolar methylene group into water corresponds to increase of ΔC_P_ by 15 cal/mol-K. Therefore, the C → O transition observed in our MD simulations results in ΔC_P_ growth by 0.12 kcal/mol-K solely due to the pore-lining residues.

### Analysis of correlated motions in MD traces

Generalized correlation coefficient based on the linear mutual information (LMI) was calculated according to the equation (1) 61:


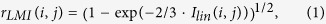


where


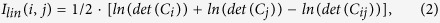


and 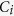, 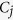, 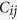
are covariance matrices for C_α_ atoms of single residues *i, j* and the couple of residues (*i, j*), respectively. Covariance matrix for C_α_ atoms of the TRPV1 was calculated using gromacs *g_covar* utility after fitting of S1–S4 backbone atoms to their initial structure.

## Additional Information

**How to cite this article**: Chugunov, A. O. *et al*. Temperature-sensitive gating of TRPV1 channel as probed by atomistic simulations of its trans- and juxtamembrane domains. *Sci. Rep.* **6**, 33112; doi: 10.1038/srep33112 (2016).

## Supplementary Material

Supplementary Information

## Figures and Tables

**Table 1:** Computations performed in this work.

ID	Starting structure, PDB code	Temperature (K)	MD length (ns); # of MD frames	System composition
C-280.1	3J5P	280	500 (2501)	4 proteins; POPC 256; POPE 121; CHOL 148; Water 43655; Cl^−^ 40
C-310.1		310	500 (2501)	
C-310.2		310	500 (2501)	
C-325.1		325	500 (5001)	
C-325.2		325	500 (5001)	
C-340.1		340	1000 (5001)	
C-340.2		340	500 (2501)	
C-340 → 310	c-340.1	310	500 (2501)	
O-280.1	3J5Q	280	500 (2501)	4 proteins; POPC 257; POPE 119; CHOL 150; Water 43561; CL^−^ 40
O-310.1		310	1000 (5001)	
O-310.2		310	500 (2501)	
