# Supplementary material for: Temperature-sensitive gating of TRPV1 channel as probed by atomistic simulations of its trans- and juxtamembrane domains
Source: Sci Rep. 2016 Sep 9;6:33112. doi: 10.1038/srep33112 (PMC5017144; doi:10.1038/srep33112)
Supplement: Supplementary Information [file srep33112-s1.pdf]

Supporting information

# Temperature-sensitive gating of TRPV1 channel as probed by atomistic simulations of its trans- and juxtamembrane domains

Anton O. Chugunov[1,*], Pavel E. Volynsky[1], Nikolay A. Krylov[1,2], Dmitry E. Nolde[1,2], Roman G. Efremov[1,3]

[1] M.M. Shemyakin & Yu.A. Ovchinnikov Institute of Bioorganic Chemistry, Russian Academy of Sciences, ul. Miklukho-Maklaya, 16/10, Moscow 117997.

[2] Joint Supercomputer Center, Russian Academy of Sciences, Leninsky prospect, 32a, Moscow 119991, Russia.

[3] National Research University Higher School of Economics, Myasnitskaya ul. 20, 101000 Moscow, Russia.

* Corresponding author. E-mail: batch2k@yandex.ru.

Keywords: TRP receptors, TRPV1, Thermosensation, Ion channel gating, Computer simulations, Membrane structure and dynamics

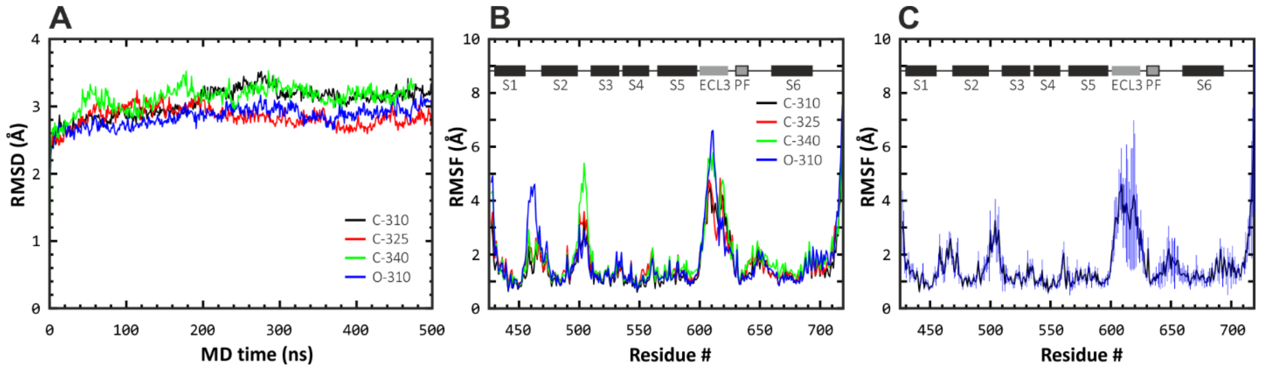


**Figure S1. Basic analysis of TRPV1 MD trajectories.** A. RMSD over the transmembrane domain for the first 500 ns from the starting structures. Four trajectories are shown according to the legend. B. A per-residue RMSF parameter for the whole model of TRPV1. Peaks correspond to loops that connect transmembrane segments. Membrane topology is shown schematically in the top of the panel. C. RMSF for individual C-310 trajectory with standard deviation (*blue lines*) over four subunits of TRPV1 channel. Note the largest s.d. for the ECL-3 region.

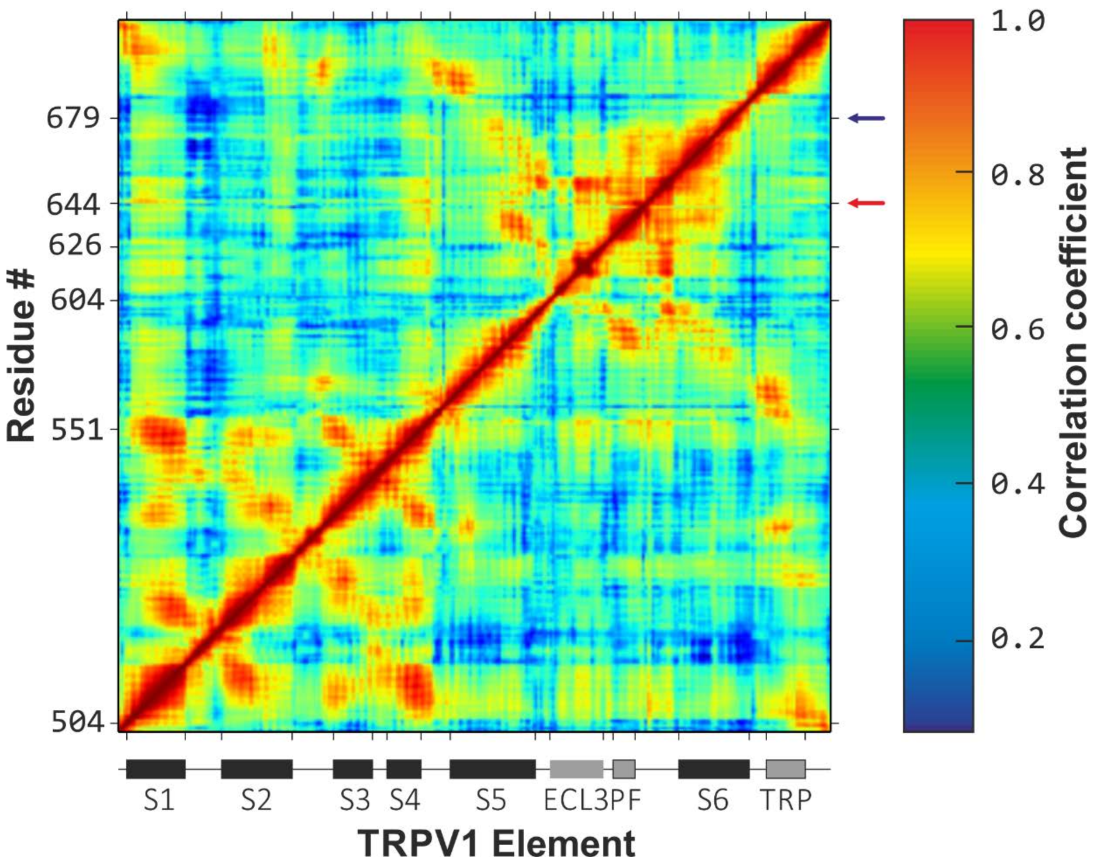


**Figure S2. Color map illustrating generalized correlation between motions of residues of one of TRPV1 monomers in MD trajectory C-325.** X-axis is subscribed with the secondary structure elements (black and grey rectangles correspond to transmembrane helices and other structural elements, respectively), Y-axis indicates the residue numbers. *Red* and *blue arrows* to the right from the map show sections of the map at the levels of Met 644 and Ile 679, respectively, that are shown in Figs. 7*B* and 7*C*.

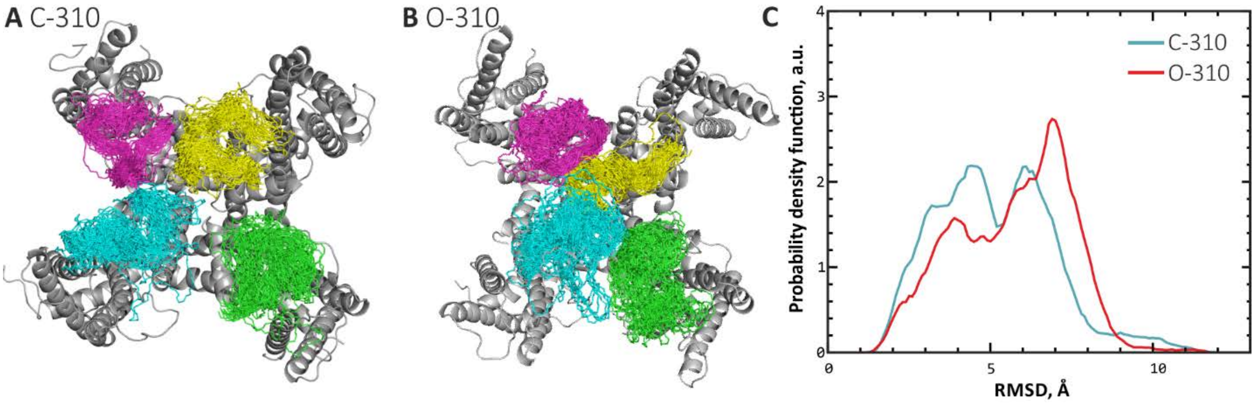


**Figure S3. Conformational dynamics of TRPV1 loop ECL-3 in closed (A) and open (B) states.** A, B. A snapshot of TRPV1 with sampling of ECL-3 conformations from MD trajectories C-310 (A) and O-310 (B). ECL-3 from each monomer is individually colored. C. Distributions of pairwise RMSDs between each pair of ECL-3 conformations in A and B. Note that O-310 distribution is shifted to the right; therefore, in the open state this loop samples a wider conformational space and has larger conformational entropy.

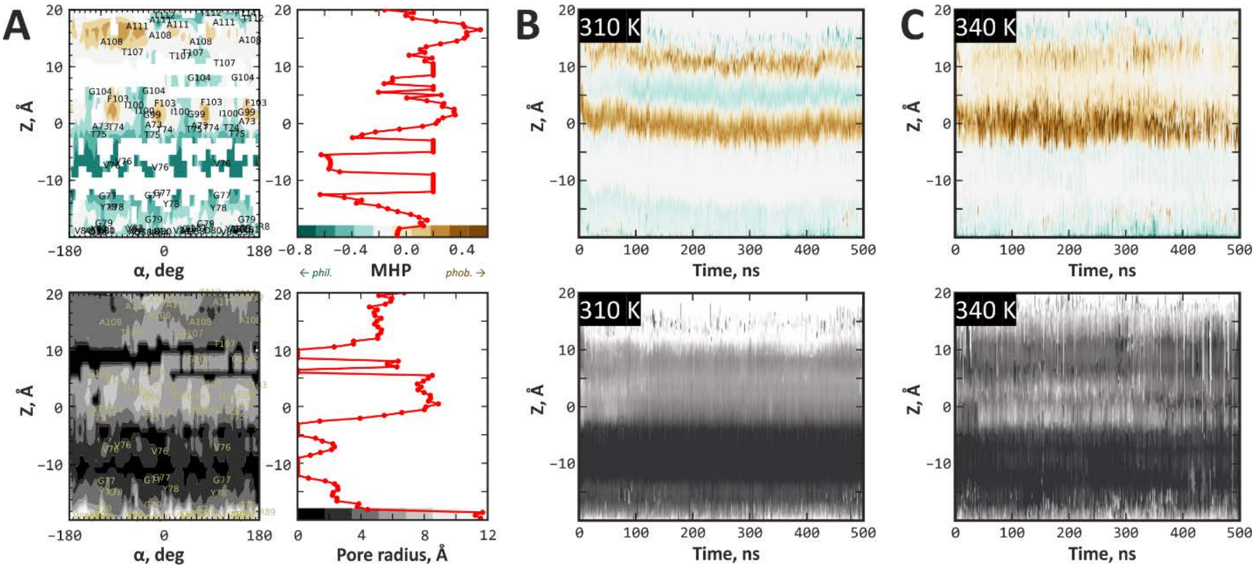


**Figure S4. Potassium channel KcsA does not open when heated.** As a negative control for TRPV1 opening in MD when heated over it's temperature activation threshold, we performed analogous MD simulation with KcsA channel that does not exhibit thermal activation. In MD, it remained stably closed at 310 and 340 K. A. Mapping of KcsA pore in the closed state (analogously to Fig. 2B). B, C. Dynamic MHP (*top*) and pore radius profiles (*bottom*) for KcsA MD at 310 K (B) and 340 K (C). Such dynamic maps are analogous to those in Figs. 3 and 5.

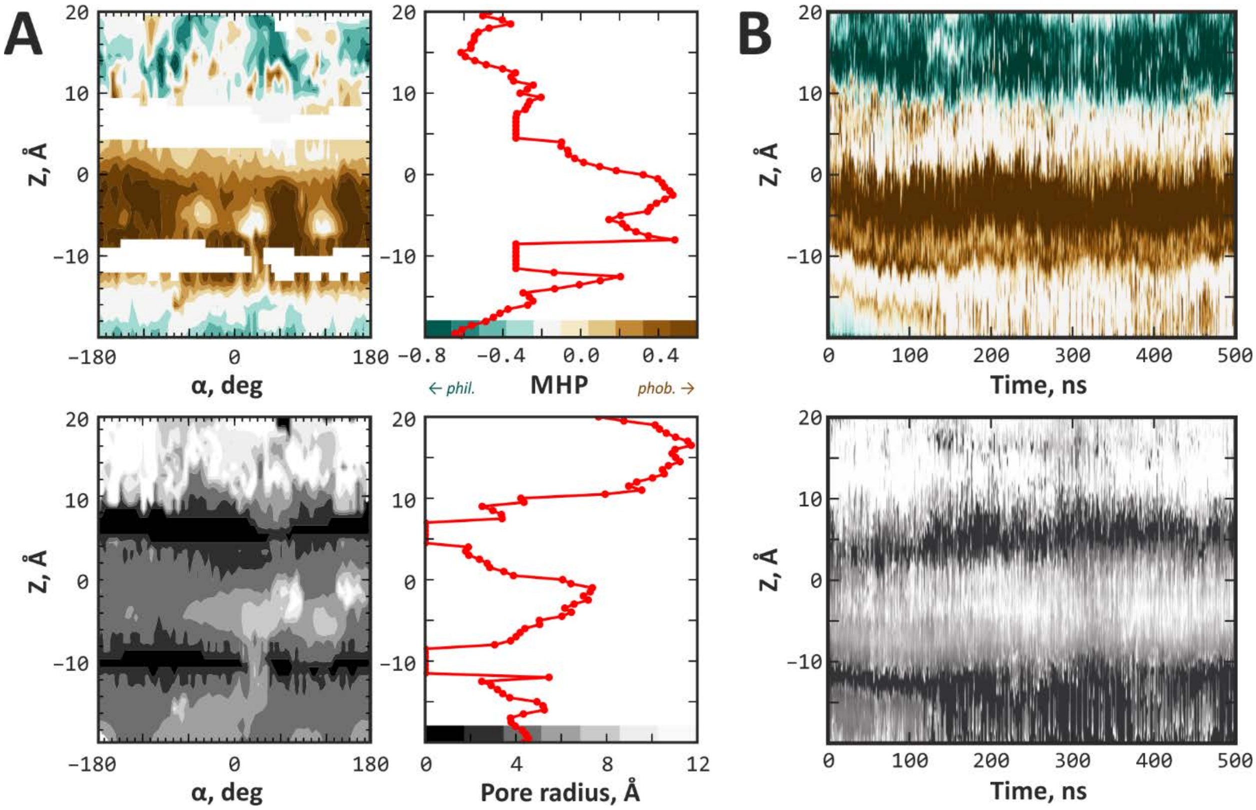


**Figure S5. Analysis of TRPV1 dynamics in DPPC bilayer at 340 K.** To assess whether the choice of the membrane lipids affects TRPV1 thermal opening process, we performed analogous to C-340 MD simulation in the saturated phospholipid (DPPC) membrane at 340 K. Initial structure was the closed channel. Analysis revealed no pore opening events. A. Mapping of TRPV1 pore analogous to Fig. 2B. B. Dynamic profiles of pore MHP (*top*) and pore radius (*bottom*) profiles, analogous to Figs. 3 and 5.

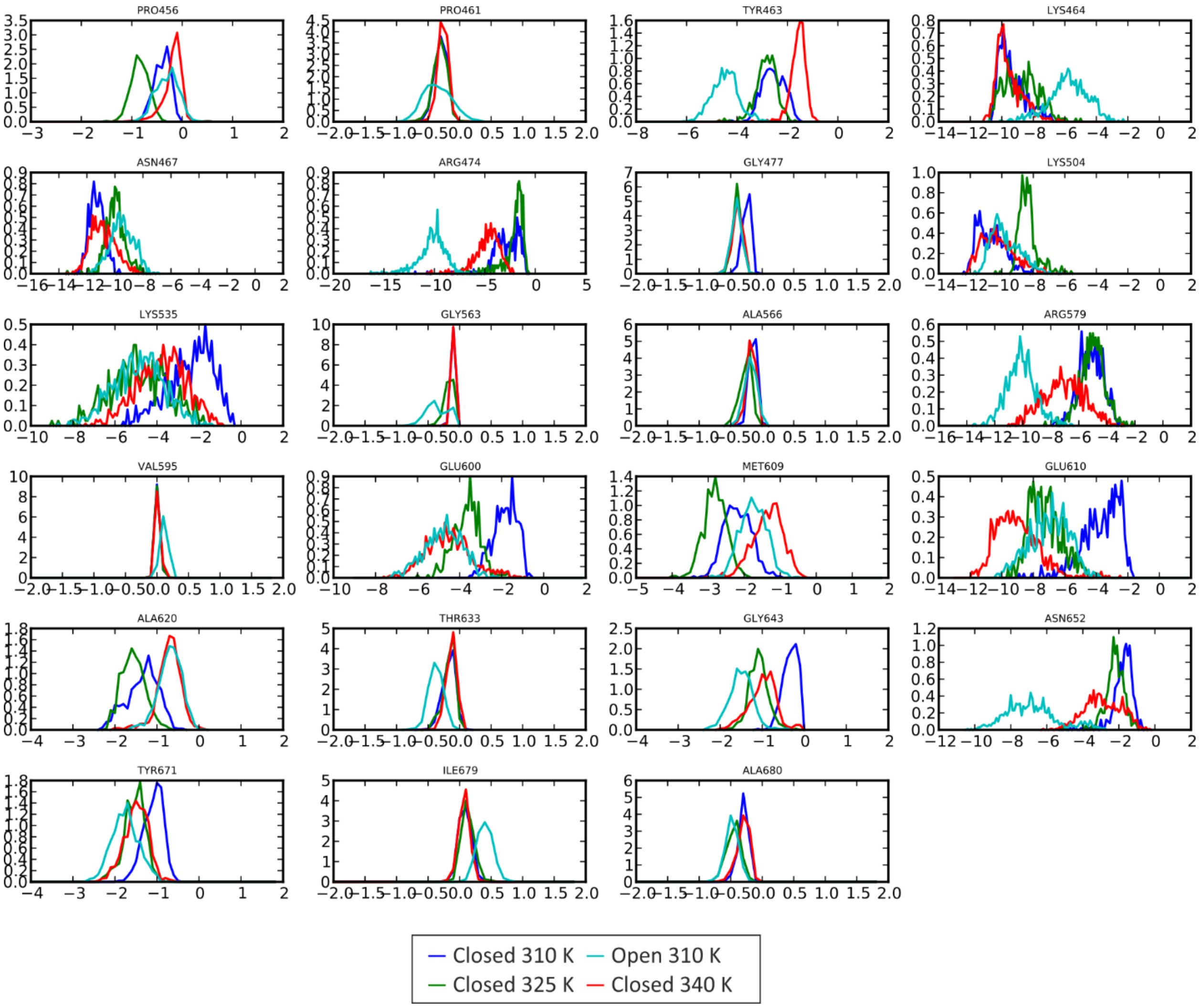


**Figure S6. TRPV1 residues that change their solvation energy most significantly during closed→open transition.** Each panel contains MD-distributions of solvation energy for four trajectories (see *Legend*). X-axis: Solvation energy (kcal/M); Y-axis: probability density function (a.u.). Among these residues, only four line the pore: Gly 643, Tyr 671, Ile 679 and Ala 680.

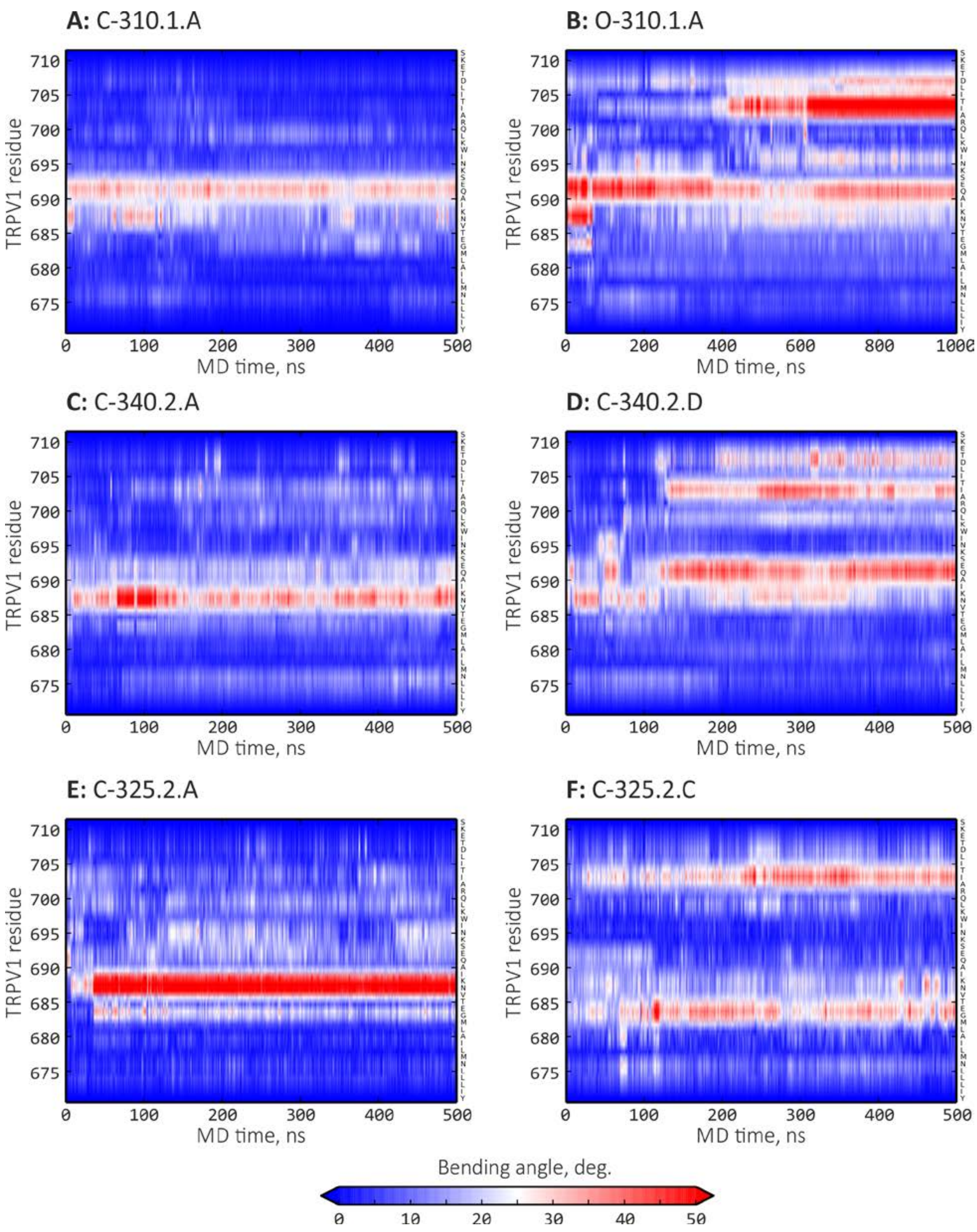


**Figure S7. Bending angle analysis of the helix S6—TRP domain (fragment 671–712) for different MD trajectories** (A: subunit A in C-310.1; B: subunit A in O-310.1; C: subunit A in C-340.2; D: subunit D in C-340.2; E: subunit A in C-325.2; F: subunit C in C-325.2). X-axis is MD time; Y-axis is the residue number; color represents the helix bending angle[1] according to the scale given in the bottom.

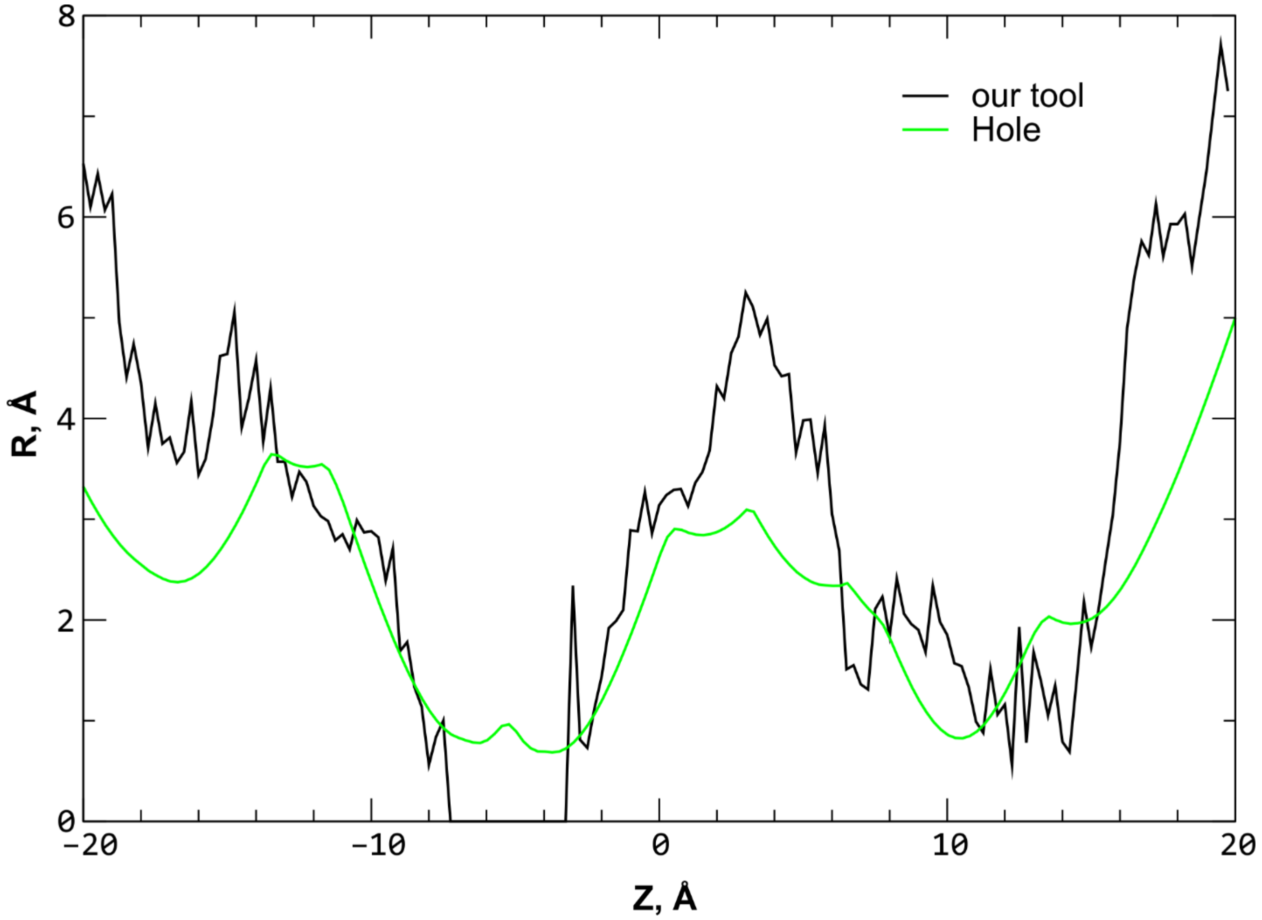


**Figure S8. Performance of our method for mapping channels' pores and the HOLE[2] approach.** The plotted parameter is pore radius (the main feature that HOLE calculates) for TRPV1 "closed" experimental cryo-EM structure (pdb ID: 3J5P). Correlation coefficient for the two plots is 0.82.

## References

1. Dahl, A.C., Chavent, M. & Sansom, M.S. Bendix: intuitive helix geometry analysis and abstraction. *Bioinformatics* **28**, 2193-4 (2012).
2. Smart, O.S., Neduvelil, J.G., Wang, X., Wallace, B.A. & Sansom, M.S. HOLE: a program for the analysis of the pore dimensions of ion channel structural models. *J Mol Graph* **14**, 354-60, 376 (1996).
